# Anti-Analgesic Effect of the Mu/Delta Opioid Receptor Heteromer Revealed by Ligand-Biased Antagonism

**DOI:** 10.1371/journal.pone.0058362

**Published:** 2013-03-15

**Authors:** Laura Milan-Lobo, Johan Enquist, Richard M. van Rijn, Jennifer L. Whistler

**Affiliations:** 1 Ernest Gallo Clinic and Research Center, University of California San Francisco, Emeryville, California, United States of America; 2 Department of Neurology, University of California San Francisco, Emeryville, California, United States of America; Hokkaido University, Japan

## Abstract

Delta (DOR) and mu opioid receptors (MOR) can complex as heteromers, conferring functional properties in agonist binding, signaling and trafficking that can differ markedly from their homomeric counterparts. Because of these differences, DOR/MOR heteromers may be a novel therapeutic target in the treatment of pain. However, there are currently no ligands selective for DOR/MOR heteromers, and, consequently, their role in nociception remains unknown. In this study, we used a pharmacological opioid cocktail that selectively activates and stabilizes the DOR/MOR heteromer at the cell surface by blocking its endocytosis to assess its role in antinociception. We found that mice treated chronically with this drug cocktail showed a significant right shift in the ED_50_ for opioid-mediated analgesia, while mice treated with a drug that promotes degradation of the heteromer did not. Furthermore, promoting degradation of the DOR/MOR heteromer after the right shift in the ED_50_ had occurred, or blocking signal transduction from the stabilized DOR/MOR heteromer, shifted the ED_50_ for analgesia back to the left. Taken together, these data suggest an anti-analgesic role for the DOR/MOR heteromer in pain. In conclusion, antagonists selective for DOR/MOR heteromer could provide an avenue for alleviating reduced analgesic response during chronic pain treatment.

## Introduction

Delta and mu opioid receptors (DOR and MOR respectively) are inhibitory G-protein-coupled receptors that regulate pain transmission. Opioids are key medications for the treatment of pain, and agonists at both the MOR and DOR are analgesics. Recently, it was shown that thermal nociception is primarily modulated by MORs while mechanical nociception is primarily mediated by DOR [1], suggesting that these receptors are expressed in distinct circuits. However, several lines of evidence also indicate that the MOR and DOR modulate one another’s function *in vivo* [2], [3], [4], and there is mounting evidence that they can form heteromers with unique properties not only *in vitro* [5], [6], [7], [8], [9], [10], but also *in vivo* [11], [12]. Moreover, a recent study revealed that the MOR crystallizes as a dimer with an extensive dimer interface comprised of TM5 and TM6 that display a high degree of homology with the DOR, again suggesting the likelihood of DOR/MOR heteromers [13]. However, deciphering the functional role of the DOR/MOR heteromer in pain transmission has been hindered by the lack of pharmacological agents selective for the heteromer over the MOR and/or DOR homomers.

Recently, we demonstrated that a mixture of the MOR agonist methadone and the DOR antagonist naltriben (NTB) has distinct pharmacological properties at DOR/MOR heteromers compared to at MOR or DOR homomers in a heterologous system [10]. Specifically, we demonstrated that a cocktail of methadone and a low dose of NTB blocked the endocytosis of the DOR/MOR heteromers but not MOR homomers, and did so without blocking signal transduction from DOR/MOR heteromers. Thus, this cocktail acts as a biased antagonist for trafficking but not signaling of the DOR/MOR heteromer, and, consequently, stabilizes functionally active DOR/MOR heteromers at the cell surface. In contrast, methadone alone (in the absence of NTB) promotes rapid endocytosis and degradation of the DOR/MOR heteromers but endocytosis and recycling of MOR homomers – in effect destabilizing the expression of DOR/MOR heteromers. We also showed that another DOR antagonist naltrindole (NTI), not only blocked endocytosis of the DOR/MOR heteromer, but also prevented its activation, suggesting that NTB and NTI show distinct effects with regard to the DOR/MOR heteromers.

There is currently no available technology to specifically isolate the action of a class A GPCR dimer from that of the homomer/monomer *in vivo*. Additionally, the lack of selective ligands for DOR/MOR heteromers has, likewise, complicated attempts to elucidate the functional role of DOR/MOR heteromers in antinociception. Specifically it is unclear whether DOR/MOR heteromers oppose the action of the homomers/monomers or have the same functional effect. Based on our previous *in vitro* findings, we devised a series of experiments designed to block downregulation of DOR/MOR heteromers *in vivo*, and, thereby, gain insight into their functional role in antinociception. The experiments here were specifically designed to determine whether stabilization of the heteromer created better antinociception, indicating an analgesic role of the heteromer, or reduced antinociception, indicative of an anti-analgesic role for the heteromer. We found that stabilization of the DOR/MOR heteromer produced reduced antinociception seen as a dramatic right shift in the dose response to a MOR agonist. This “anti-analgesic” effect was reversed by either blocking signaling from the DOR/MOR heteromer or by promoting its endocytosis and degradation.

## Materials and Methods

### Ethics Statement

All animal procedures in this study were approved by the Gallo Center Institutional Animal Care and Use Committee and were conducted in agreement with the Guide for the Care and Use of Laboratory Animals (National Research Council, 1996) in our AAALAC certified facility.

### Reagents

Naltriben mesylate (NTB), (+)-4-[(αR)-α-((2S,5R)-4-allyl-2,5-dimethyl-1-piperazinyl)-3-methoxybenzyl]-N,N-diethylbenzamide (SNC80) and D-Phe-Cys-Tyr-D-Trp-Arg-Thr-Pen-Thr-NH2 (CTAP) were purchased from Tocris (Ellisville, MO). (±)-Methadone hydrochloride and Naltrindole hydrochloride (NTI) were purchased from Sigma-Aldrich (St. Louis, MO). Compounds were dissolved in water or saline, with the exception of NTB and NTI, which were dissolved in 5% dimethylsulfoxide (DMSO). Mouse M1 and M2 monoclonal antibody, anti-FLAG M2 affinity matrix, albumin from bovine serum, L-glutathione, iodoacetamide, Triton X-100 and Tween 20 were purchased from Sigma (St. Louis, MO). Anti-HA.11 beads were from Covance.

### Cell Culture

HEK293 cells (American Type Culture Collection) were grown in Dulbecco’s modified Eagle’s medium (Life Technologies, CA) supplemented with 10% fetal bovine serum (Thermo Scientific HyClone, UT). N-terminal signal sequence and either HA- or FLAG-tagged c-DNAs of the murine opioid receptor constructs were stably expressed in HEK293 cells. For generation of clonal stable cell lines, single colonies were chosen and propagated in the presence of selection-containing medium. Cell lines were carefully matched for expression (see [14]).

### Biotin Protection Endocytosis and Endocytosis-degradation Assays

HEK293 cells stably expressing N-terminal FLAG-MOR alone or FLAG-MOR and HA-DOR together were grown to 90% confluency in 10-cm plates. Cells were washed twice in PBS and biotinylated with 0.3 mg/ml disulfide-cleavable biotin (Pierce, Rockford, IL) at 4°C for 30 minutes to selectively label a pool of receptors at the cell surface as described in [15]. For quantification of **endocytosis**, cells were washed in PBS and placed in pre-warmed medium for 15 minutes before treatment with ligand or vehicle (no treatment) for 30 minutes. For quantification of **stability/degradation**, cells were incubated with ligand for prolonged periods of time as indicated. Concurrent with ligand treatment “total” and “strip” plates remained at 4°C. After ligand treatment, plates were washed in PBS, and remaining cell surface-biotinylated receptors were stripped twice in 50 mM glutathione, 75 mM NaCl, 75 mM NaOH, 10% fetal bovine serum at 4°C, for 60 minutes (strip was done in all plates with the exception of the “total” plate). Cells were quenched with PBS containing 50 mM iodoacetamide, 10% bovine serum albumin for 30 minutes (including “total” plate). Afterward, all cells were lysed in 0.1% Triton X-100, 150 mM NaCl, 25 mM KCl, 10 mM Tris·HCl, pH 7.4, with protease inhibitors (Roche Applied Science, Basel, Switzerland). Lysates were cleared by centrifugation at 10,600 g (Eppendorf rotor 5417R) for 10 minutes at 4°C. In cells expressing only one type of receptor, lysates were incubated overnight at 4°C with anti-FLAG M2 or HA.11 affinity matrix (depending on the epitope tag), washed and resolved by SDS-PAGE. The “protected” pool of endocytosed receptors were visualized by streptavidin overlay. This protected pool of biotinylated receptor shrinks over time for receptors that are degraded, since no new receptors are biotinylated. In contrast the protected pool remains constant for receptors that are endocytosed, recycled and re-endocytosed. For monitoring homomer versus heteromer trafficking in the same cells, cells were biotinylated, treated with agonist for the indicated time, stripped, quenched and lysed as above. Lysates were then incubated with anti-FLAG M2 affinity matrix, overnight at 4°C which immunoprecipitates both FLAG-MOR homomers and FLAG-MOR/HA-DOR heteromers. The lysate remaining was separated from the pellet and then immunoprecipitated with HA.11 affinity matrix to isolate HA-DOR homomers. The pellet containing FLAG M2 affinity matrix, and therefore both MOR homomers and DOR/MOR heteromers, was incubated with FLAG peptide to release all receptors to the lysate. This lysate was then incubated with HA.11 affinity matrix to selectively immunoprecipitate HA-DOR/FLAG-MOR heteromers (that had already been immunoprecipitated with M2 matrix). The HA.11 affinity matrix now contains the DOR/MOR heteromers while the lysate contains MOR homomers. Finally, the lysate remaining from the immunoprecipitation with HA.11 affinity matrix was incubated with anti-FLAG M2 affinity matrix to specifically isolate FLAG-MOR homomers. All matrix/beads were washed and the precipitates were deglycosylated with PNGase (New England Biotechnology, Beverly, MA) in 10 mM Tris, pH 7.5, for 1 h at 37°C, denatured with SDS sample buffer (no reducing agent), and resolved by SDS/PAGE. Blots were blocked in 5% milk, washed thoroughly and incubated with Vectastain ABC reagent (Vector Laboratories, Burlingame, CA) for 30 minutes and washed thoroughly again. Blots were developed with enhanced chemiluminescence reagents (ECL, GE Healthcare, NJ), scanned and quantified using ImageJ Software.

### Calcium Mobilization Assay

HEK293 cells stably co-expressing N-terminal FLAG-MOR and HA-DOR were seeded onto 96-well black clear bottom plates from Corning. Cells were then transiently transfected with chimeric G protein Δ6-G_qi4_-myr (100 ng for every 70,000 cells) [16]. One day after transfection, cells were loaded for 60 minutes with a Ca^2+^-fluorophore (Molecular Devices, Sunnyvale, CA) and stimulated with ligand as indicated in the figure legends. Cells were pre-incubated with antagonist at stated concentration for 20 minutes prior to measurement of intracellular Ca^2+^ release in a Flex-3 station apparatus in relative fluorescence units (RFU; Molecular Devices, Sunnyvale, CA) for 2 minutes. Data are represented as percentage (%) of the maximal effect given by the MOR agonist.

### Radiolig and Binding

24 hours after the last drug administration, mice were sacrificed by cervical dislocation and their spinal cord harvested for analysis of delta opioid receptors. Spinal cord tissue from each treatment (n = 20 mice) was divided into three groups of 6–7 mice to provide three independent samples to assess reproducibility of the assay. Tissue from each group was homogenized in ice cold 0.32 M sucrose-containing binding buffer (50 mM Tris-HCl, pH 7.4/1 mM EDTA/2 mM MgCl2). Samples were centrifuged at 1500 g for 15 min, 4°C, then supernatant was further centrifuge at 31,000 rpm in a Beckman 45Ti rotor for 20 min and resuspended in binding buffer. This last step was repeated twice, and the final pellet frozen at −80°C until use. Protein concentration was determined by Pierce® BCA protein assay kit (Thermo Scientific, IL).

DOR number was measured using Enkephalin, [Tyrosyl-2,6-3H(N)]-(2-D-Penicillamine, 5-D-Penicillamine) ([^3^H]-DPDPE) (43 Ci/mmol, Perkin Elmer, MA). Samples corresponding to 85 µg protein were prepared in binding buffer containing 50 mM Tris-HCl pH 7.4./1 mM EDTA/2 mM MgCl2 and 0.16 nM - 20 nM [^3^H]-DPDPE (each concentration in triplicate) in a final volume of 200 µl. Samples were incubated for 60 minutes at RT in a 96 well plate and filtered through Whatman GF/B filters. The filters were washed three times in ice cold binding buffer and dried overnight at room temperature. The filters were then incubated overnight in 50 µl of scintillation fluid (Microscint 20, Perkin Elmer) prior to counting in a Packard cell top scintillation counter (Perkin Elmer). Specific binding was calculated as total minus nonspecific binding performed in the presence of cold DADLE (1 µM).

### Analgesic Response: Tail-flick Reflex to Heat Irradiation

Wild type C57/BL6 and DOR KO mice (KO are the C57/BL6 background) were tested for antinociception using the radiant heat tail-flick procedure. Mice with robust tail-flick reflexes and baseline latencies of 2.0 through 3.5 seconds were included in the study; a maximum latency of 10 seconds was set as the “cutoff” time to minimize damage to the tail. Dose response was measured by cumulative drug addition, and nociceptive assessment 20 minutes after each subcutaneous (s.c.) administered dose, three to four doses per animal. Data is presented as percentage of maximal possible effect:

MPE = ((latency after drug - baseline)/(cutoff - baseline)) * 100.

### Mechanical Sensitivity

One day prior to testing, mice were placed in plastic chambers on a wire mesh grid to habituate for one hour. On test day, mice were placed in the chambers one hour before injection. Prior to injection a baseline measurement was performed. Mechanical sensitivity was measured by stimulating the plantar surface of the hind paw of the mouse with von Frey filaments (0.04, 0.07, 0.16, 0.4, 0.6, 1, 1.4 and 2 g). The largest filament (2 g) was used as cutoff. The lowest force that evoked a paw withdrawal response in two out of three tests was recorded. Both paws were measured and the average was used for each animal. Data is represented as percentage of maximal possible effect (MPE) which is defined as ((measurement - baseline)/(cutoff – baseline)) * 100.

### Statistical Analysis

Dose response curves were calculated using GraphPad (San Diego, CA) Prism software with a linear regression and 95% confidence intervals (CI) of X when Y = 50. When ED_50_ values were compared, all of the data were analyzed together, and values with separate, not overlapping, 95% confidence intervals at p<0.05 were considered significantly different.

## Results

### A Low Dose of the DOR Antagonist NTB does not Alter Acute Antinociception to the MOR Agonist Methadone in Wild Type Mice

**Figure 1:**
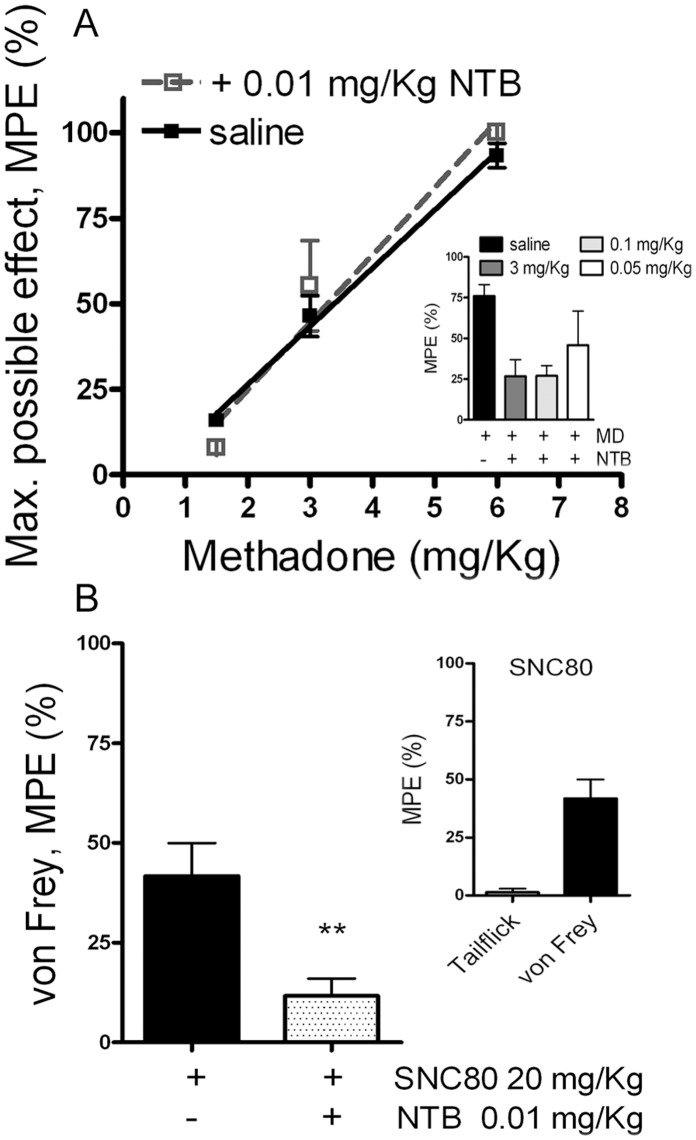
Low dose of the DOR antagonist naltriben (NTB) does not affect acute thermal antinociception to the MOR agonist, methadone, but is sufficient to block the effect of the DOR agonist, SNC80, on mechanical sensitivity. A) Acute antinociceptive response was measured by tail-flick in C57/BL6 wild type mice after escalating doses (s.c.) of methadone alone (closed squares) or in combination with a low dose of NTB (0.01 mg/Kg; open squares); n = 10 mice in both groups. Inset (A) shows acute methadone antinociception in the presence of higher doses of NTB (3 mg/Kg and 0.1 mg/Kg); n = 10 mice for each NTB concentration. B) Acute mechanical sensitivity was measured by von Frey assay in C57/BL6 wild type mice after 20 mg/Kg of SNC80 (s.c.) alone or in combination with 0.01 mg/Kg of NTB. Inset (B) shows the selective mechanical antinociceptive effect of SNC80. Data represents mean ± SEM; n = 10 mice per concentration. (Unpaired-t test, p = 0.005).

Consistent with its properties as a DOR-selective antagonist, NTB at low doses had no effect on antinociception produced by the MOR agonist methadone (Fig. 1A), but blocked antinociception produced by the DOR-selective agonist SNC80 (Fig. 1B). At higher doses, NTB lost selectively and could antagonize methadone-mediated antinociception (Fig. 1A inset). Therefore, all experiments were carried out at doses of NTB that did not affect methadone antinociception acutely.

### Continuous Activation and Stabilization of DOR/MOR Heteromers Reduces Methadone-mediated Antinociception

**Figure 2:**
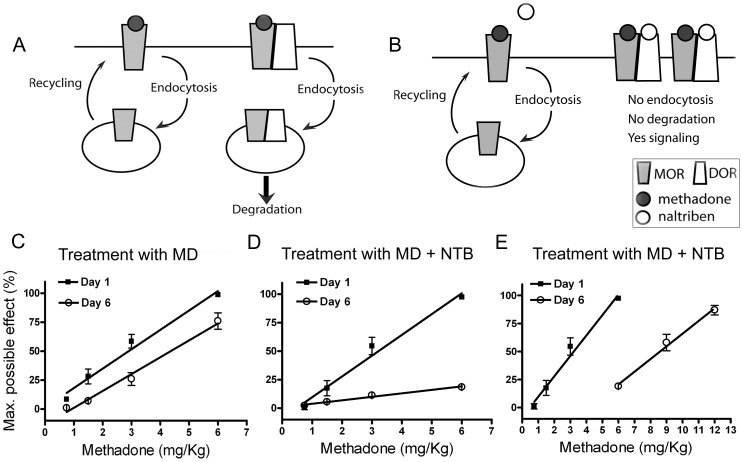
Development of reduced antinociception after chronic treatment with a cocktail of methadone and NTB. A & B) Proposed model of the trafficking of MOR and DOR/MOR in response to methadone (A) or to methadone/NTB cocktail treatment (B); MOR will be activated, internalized and recycled back to the plasma membrane in response to methadone. Normal cycling will keep the MOR ready for further activation. DOR/MOR will be activated, internalized and degraded in response to methadone. In the presence of the DOR antagonist NTB, activation and trafficking of MOR in response to methadone will remain unaffected, whereas DOR/MOR heteromers will be occupied by NTB and methadone resulting in the activation of the receptor complex without subsequent endocytosis and degradation. C–E) Antinociception to escalating doses of methadone was measured in naïve wild type mice on day 1 (closed squares). ED_50_ values calculated via linear regression analysis and 95% confidence intervals are as follows: Day1, MD treatment: 3 (1.9–3.8) mg/Kg and MD+NTB treatment: 3.2 (2.3–4.2) mg/Kg. On days 2, 3, 4 and 5, mice were injected s.c. once daily with the ED_50_ dose of methadone (3 mg/Kg) (C) or a cocktail of methadone (3 mg/Kg) combined with NTB (0.01 mg/Kg) (D). On day 6 (open circles), antinociception to methadone was measured again in mice treated with only methadone (C) or the cocktail (D); ED_50_ values and 95% confidence intervals are as follows: Day 6, MD treatment: 4.3 (3.6–5.3) mg/Kg and MD+NTB treatment: 8.6 (5.4–12.4) mg/Kg. E) Shows an additional dose range of methadone on day 6 for the group of mice receiving injections of methadone/NTB cocktail. Data represents mean ± SEM; n = 20 mice per group.

Previously, we demonstrated *in vitro* that selective doses of NTB produce biased antagonism on DOR/MOR heteromers activated by methadone, whereby it selectively antagonizes endocytosis but not signal transduction from the DOR/MOR heteromer ([10], and see Figure S1A, B). Because the DOR/MOR heteromer is rapidly degraded after endocytosis in response to methadone alone ([10], and see Figure S1C), we hypothesized that treatment with methadone alone would favor signaling from MOR homomers (hypothesis cartooned in Fig. 2A) while co-treatment with methadone and NTB would stabilize the DOR/MOR heteromer (hypothesis cartooned in Fig. 2B), and thereby allow an assessment of the functional contribution of this heteromer to antinociception. Specifically, we hypothesized that if DOR/MOR heteromers (like MOR homomers) are anti-nociceptive, stabilizing this target would enhance analgesia across time. In contrast, if DOR/MOR heteromers oppose the action of MORs for analgesia, stabilization of this target over time would reduce the analgesic effect of methadone.

To examine these hypotheses, we monitored the ED_50_ of methadone before and after chronic treatment with either methadone alone or a cocktail of methadone plus NTB. First, to establish the initial ED_50_ for methadone, all mice (n = 40) were treated with accumulative doses of methadone (0.75, 1.5, 3, 6 and 9 mg/Kg) until 100% of maximal possible effect (MPE) for antinociception was achieved (Figure 2C, D & E; Day 1, closed squares). Mice were then divided into two groups (n = 20 per group). One group received an injection of methadone only (ED_50_ dose; 3 mg/Kg), once per day for 5 days. The second group received an injection of methadone (3 mg/Kg) mixed with NTB (0.01 mg/Kg, a dose that has no effect on acute antinociception, see Fig. 1A). On day 6, the ED_50_ for methadone was measured once again (Figure 2C, D & E; Day 6, open circles) and compared with that on day 1. Mice treated with methadone only, showed a 1.4x fold right shift in the ED_50_ for methadone (Fig. 2C, ED_50_ with 95% confidence intervals (CI): 3.0 (1.9–3.8) and 4.3 (3.6–5.3) mg/Kg for day 1 and day 6 respectively. Similar shifts in ED_50_ have been previously described after treatment with moderate doses of methadone (see Table 1 in [17] with similar shift in ED_50_ of wild type mice, and see [18]). In contrast, mice co-administered methadone and NTB showed a 2.7x fold shift in the ED_50_ for methadone on day 6 (Figure 2D & E, ED_50_ with 95% CI: 3.2 (2.3–4.2) and 8.6 (5.4–12.4) mg/Kg for day 1 and day 6, respectively), indicating a significant decrease in methadone-mediated analgesia. An additional cohort of mice (n = 19) was treated with only 0.01 mg/Kg of NTB once daily for 5 days to control for the effects of NTB alone. We also observed a 1.7x fold right shift in ED_50_ between day 1 and day 6 (ED_50_ with 95% CI: 3.1 (0.3–8.1) and 5.2 (3.9–6.8) mg/Kg respectively), similar to what occurred in the methadone only group. Together these results indicate that a combination of the agonist methadone with the antagonist NTB is necessary for the dramatic right shift in methadone-mediated analgesia shown in Figure 2D & E.

### Treatment with Methadone Alone Reverses the Right Shift in Analgesia Induced by Chronically Administered Methadone Plus NTB

**Figure 3:**
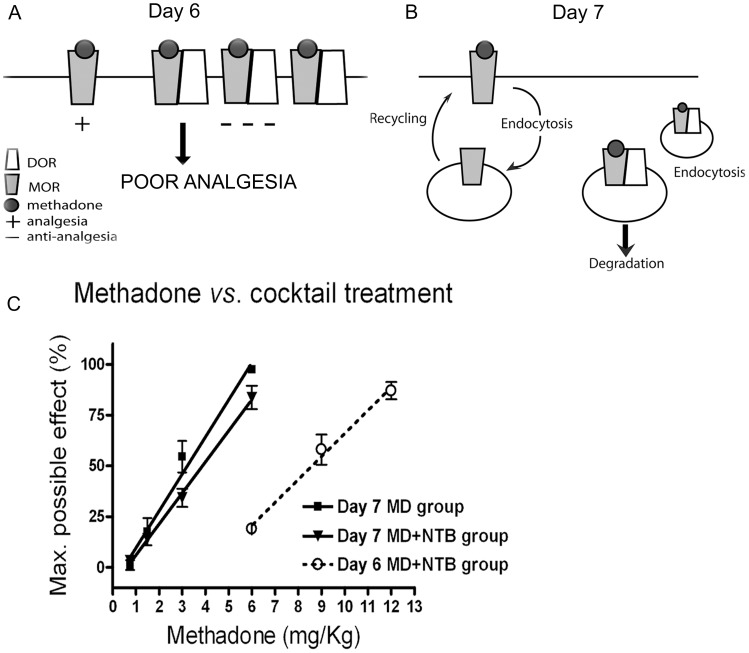
A single methadone treatment reverses the reduced antinociception produced by the methadone/NTB cocktail. A) Proposed model of reduced analgesia produced by the MOR/DOR heteromer in mice treated with methadone/NTB cocktail, where DOR/MORs are anti-analgesic and contribute to poor antinociception. B) Proposed model of a single methadone exposure on the trafficking of MOR and DOR/MOR heteromers after induction of poor antinociception. MOR on day 6 will be activated, internalized and recycled back to the plasma membrane in response to the methadone treatment. Normal cycling will keep the MOR ready for further activation. DOR/MOR on day 6 will be activated, internalized and degraded as a consequence of the methadone treatment. C) Antinociception to escalating doses of methadone on day 7, in the same mice previously shown in Figure 2C & D (closed squares for methadone group: ED_50_ and 95% confidence intervals: 4.5 (3.4–6.7) mg/Kg, and closed triangles for cocktail group: ED_50_ and 95% confidence intervals: 3.8 (3.5–4.2) mg/Kg). Open circles show antinociception to methadone on day 6 in the cocktail group: ED_50_ and 95% confidence intervals: 8.6 (5.4–12.4) mg/Kg. Data represents mean ± SEM; n = 20 mice per group.

Based on our previous work [10], we hypothesized that chronic treatment with methadone and NTB stabilized DOR/MOR heteromers at the cellular surface. Based on Figure 2, we further hypothesized that these DOR/MOR heteromers contribute negatively to antinociception (see cartoon in Fig. 3A), because of the significant right shift in the ED_50_ for methadone after the chronic cocktail treatment (Fig. 2D & E). If this were the case, then treatment with methadone alone after cocktail treatment, should promote endocytosis and degradation of the DOR/MOR heteromer [10], and thereby shift the methadone analgesic response back to the left (see hypothesis cartooned in Fig. 3B). To test this hypothesis, we assessed analgesia to methadone in mice treated with cocktail (or methadone alone) for five days and then methadone alone on day 6. Analgesia was measured on day 7, 24 hours after the last injection with methadone alone. Mice received accumulative doses of methadone on day 6 and day 7 to assess the shift in ED_50_ (0.75, 1.5, 3, 6 and 9 mg/Kg) (Figure 3C; Day 7, closed squares methadone only mice and closed triangles for cocktail of methadone plus NTB mice; Day 6, open circles for cocktail of methadone plus NTB mice). As expected, mice that received the cocktail of methadone plus NTB showed a right shift analgesia to methadone on day 6 (Fig. 2D, E and Fig. 3C open circles). However, on day 7, 24 hours after treatment with methadone alone, analgesia to methadone was shifted 2.3x fold back to the left in this group of mice (Figure 3C, closed triangles *vs.* open circles, ED_50_ with 95% CI: 8.6 (5.4–12.4) mg/Kg on day 6 compared to 3.8 (3.5–4.2) mg/Kg shown on day 7). In contrast, there was no change in the ED_50_ or % of MPE to methadone on day 6 versus day 7 in mice chronically treated with methadone alone on all days (Figure 3C, closed squares; ED_50_ with 95% CI: 4.3 (3.6–5.3) mg/Kg on day 6 compared to 4.5 (3.4–6.7) mg/Kg on day 7).

**Figure 4:**
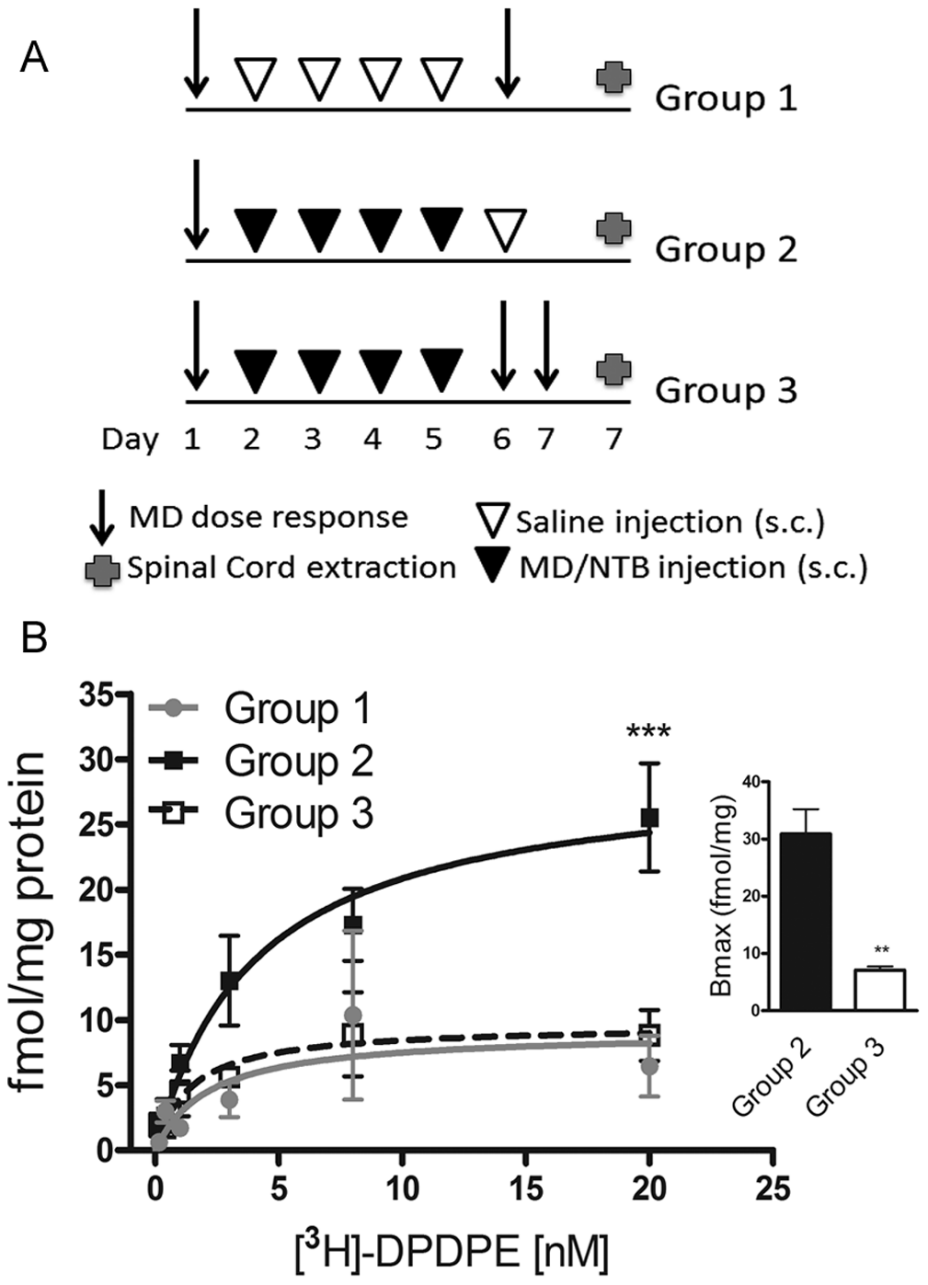
Reduced delta opioid receptor number in methadone/NTB cocktail-treated mice that were injected with methadone alone on day 6. A) Three different cohorts of mice (n = 10 for group 1, n = 20 each group 2 & 3) received accumulating doses of methadone to measure analgesia. Then, group 1 was treated with once daily injection of saline whereas group 2 and 3 were treated with a cocktail of methadone (3 mg/Kg) combined with NTB (0.01 mg/Kg) for 5 days. On day 6, only group 1 and 3 received accumulating doses of methadone to measure analgesia while group 2 received saline. Mice treated with the cocktail of MD/NTB (group 2) showed an increased number of DOR binding sites assessed by saturation binding using [^3^H]-DPDPE compared to mice treated with cocktail and challenged with methadone alone on day 6 (group 3). Mice treated with the cocktail of MD/NTB and challenged with methadone alone on day 6 (group 3) showed DOR levels comparable to the saline treated mice. Radioligand binding experiments were carried out in triplicates. Data shown for groups 2 & 3 are mean ± SEM fmol [^3^H]-DPDPE bound per mg of protein from three independent groups of 6–7 mice per group (two-way ANOVA and Bonferroni post-test: ***p<0.001). Inset represents the mean ± SEM of the Bmax from the three independent groups of mice in groups 2 & 3 (Unpaired t-test, p = 0.005). Radioligand binding data from the saline treatment (group 1) represents mean ± SEM of one group of 10 mice, carried out in triplicate.

We hypothesized that this left shift was due to endocytosis and degradation of anti-analgesic DOR/MOR heteromers in response to methadone (see cartoon Fig. 3A). To examine this possibility, we assessed the number of functional DOR binding sites by saturation binding of spinal cord membranes from mice treated with methadone plus NTB (Fig. 4, closed squares, labeled group 2) and those treated with methadone plus NTB and then methadone alone on day 6 (Fig. 4, open squares, labeled group 3). Indeed, treatment with methadone alone on day 6 caused a significant downregulation of DOR binding sites (Bmax 29.3±4.1 fmol/mg from mice treated with cocktail that did not receive methadone on day 6 *versus* Bmax 9.6±1.6 fmol/mg from mice treated with cocktail that received methadone on day 6). DOR binding sites in spinal cord were measured in a separate cohort of mice chronically treated with saline for 5 days (Fig. 4B, closed circles, labeled group 1) to establish the baseline levels of DORs in mouse spinal cord. Saline treated mice had a Bmax of 9.1±3.3 fmol/mg and showed the same ED_50_ of methadone before and after chronic treatment (Fig. S2). These data suggest an upregulation of DOR binding sites after repeated injections of methadone plus NTB (Fig. 4B, closed squares) which can be reverted to baseline with a single treatment with methadone (Fig. 4B, open squares). Additionally, since methadone has a low affinity for DOR, and does not promote DOR endocytosis even at saturating concentration (see *in vitro* results in [10]), these data suggest that the fewer DOR binding sites measured in group 3 likely represent DORs dimerized with MORs, where methadone binds and promotes endocytosis. Taken together, our data suggest that downregulation of DOR/MOR heteromers in response to methadone could reverse the right shift in dose response produced by the cocktail of methadone plus NTB *in vivo* (Figure 3C).

### Naltrindole, a DOR Antagonist that Blocks Signaling from the DOR/MOR Heteromer, Reverses the Right Shift in the Analgesia Dose Response Induced by the Cocktail of Methadone Plus NTB

Mice with a disruption of the DOR (DOR KO) did not show any difference (1.2x fold) in methadone-mediated analgesia before and after chronic cocktail methadone plus NTB treatment (ED_50_ with 95% CI: 4.4 (3.2–5.7) and 5.0 (3.5–7.2) mg/Kg on day 1 and day 6 respectively), suggesting that the right shift in ED_50_ observed in wild type mice is not mediated by an off target effect of NTB that is somehow unmasked by injecting methadone on test day 6 (Fig. 4A). Rather these data indicate that the effect of NTB must be mediated by an anti-analgesic effect of a receptor complex containing the DOR and responding to the MOR selective agonist methadone, in all likelihood the DOR/MOR heteromer. If this were the case, antagonizing the DOR/MOR heteromer after its upregulation should also shift the methadone dose response back to the left.

**Figure 5:**
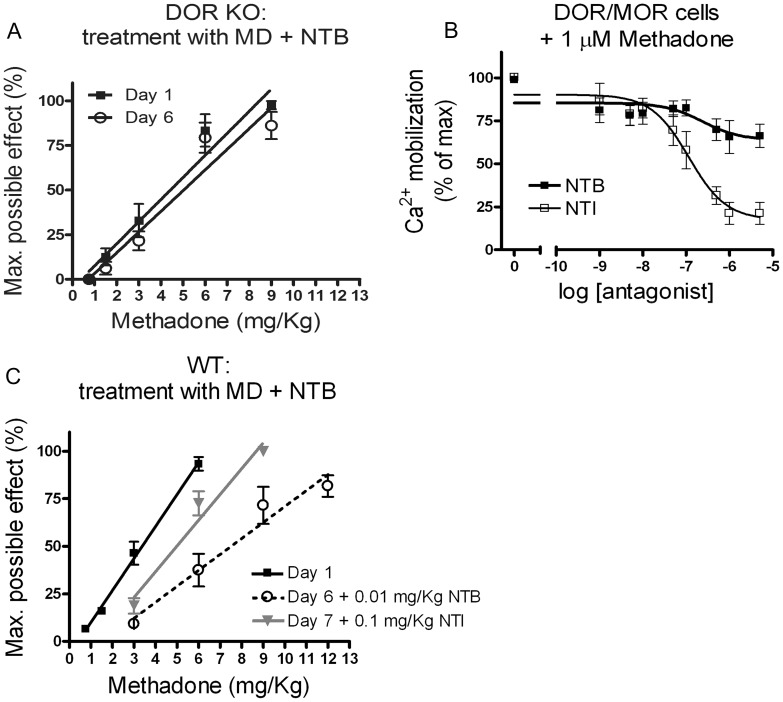
Reduced antinociception after treatment with the methadone/NTB cocktail requires the presence of DOR. Blocking signaling from the DOR/MOR heteromer reverses the analgesic effect produced by the methadone/NTB cocktail. A) Antinociception to escalating doses of methadone was measured in naïve DOR KO mice on day 1 (closed squares, ED_50_ with 95% confidence intervals: 4.4 (3.2–5.7)). On days 2, 3, 4 and 5, DOR KO mice were injected s.c. once with a cocktail of methadone (3 mg/Kg) combined with NTB (0.01 mg/Kg). Antinociception was measured on day 6 (open circles, ED_50_ with 95% confidence intervals: 5.0 (3.5–7.2) mg/Kg). Data represents mean ± SEM; n = 10 DOR KO mice with a C57/BL6 background. B) HEK293 cells co-expressing DOR and MOR were pretreated with increasing concentrations of DOR antagonist NTB (closed squares) or NTI (open squares) for 20 minutes. Intracellular calcium release (see methods) was measured after stimulation with a fixed concentration of methadone (1 µM). Data represents mean ± SEM; n = 3–4 experiments carried out in triplicate. C) Antinociception to escalating doses of methadone was measured in naïve WT mice on day 1 (closed squares, ED_50_ with 95% confidence intervals: 3.3 (3.0–3.7) mg/Kg). On days 2, 3, 4 and 5, mice were injected s.c. once with a cocktail of methadone (3 mg/Kg) combined with NTB (0.01 mg/Kg). On day 6 antinociception was measured to escalating doses of methadone combined with a fix dose of NTB (0.01 mg/Kg) to prevent DOR/MOR heteromer internalization and subsequently degradation (open circles, ED_50_ with 95% CI: 7.5 (6.2–8.6) mg/Kg). On day 7, antinociception was measured to escalating doses of methadone combined with a fixed dose of NTI (0.1 mg/Kg) to block signaling from DOR/MOR heteromer (closed triangles, ED_50_ with 95% CI: 4.9 (4.0–5.9) mg/Kg). Data represents mean ± SEM; n = 15 mice.

We have previously shown *in vitro* that NTB and another DOR-selective antagonist naltrindole (NTI) have distinct antagonist biases at the DOR/MOR heteromer. In particular, while NTB blocks only endocytosis but not signaling from the DOR/MOR heteromer, NTI blocks both endocytosis and signaling in response to methadone ([10] and see also Fig. 5B). If signaling from the DOR/MOR heteromer was responsible for the reduced antinociception produced by the methadone plus NTB cocktail, we predicted that blocking these receptors with NTI in the presence of methadone would reverse the right shift in analgesia. To test this hypothesis, analgesia to methadone was again measured in a new cohort of mice (n = 15) (Fig. 5C, closed squares; ED_50_ with 95% CI: 3.3 (3.0–3.7) mg/Kg on day 1. This cohort was then treated with the methadone plus NTB cocktail once daily for 5 days. On day 6, we confirmed a 2.3x fold right shift in analgesia with an accumulative dose response of methadone plus NTB cocktail (Fig. 5C, open circles; ED_50_ with 95% CI: 7.5 (6.2–8.6) mg/Kg on day 6). We included 0.01 mg/Kg NTB to the accumulative doses of methadone to prevent internalization and subsequently degradation of the DOR/MOR heteromer. On day 7, we repeated the dose response curve, but instead of adding NTB to the methadone, we added 0.1 mg/Kg NTI. Specifically, we chose a dose of NTI (0.1 mg/Kg) that has no acute effect on antinociception produced by the MOR agonist methadone but blocks antinociception produced by the DOR-selective agonist SNC80 (Fig. S3). In the presence of NTI, analgesia was shifted 1.5x fold back to the left (Fig. 4C, closed triangles; ED_50_ with 95% CI: 4.9 (4.0–5.9) mg/Kg on day 7). Thus, treatments that promote internalization and degradation of the heteromers (i.e. methadone alone, see Fig. 4), and antagonists that block signaling from the heteromers (i.e. NTI, see Fig. 5B) both improved antinociception after a cocktail treatment of methadone plus NTB (Fig. 3C and 5C respectively).

## Discussion

In this study, we performed a set of experiments designed to discern the functional role of the DOR/MOR heteromer in analgesia. Our studies suggest that the DOR/MOR heteromer contributes negatively to thermal analgesia. Specifically, we show that treatment with a cocktail of methadone and NTB that stabilizes heteromers at the surface while allowing signal transduction (Fig. S1), dramatically shifts the dose response to methadone to the right (Fig. 2). Furthermore we show that a single treatment with a drug that promotes degradation of the heteromer (methadone alone, Fig. 3 & 4), or treatment with a drug that blocks signaling from the heteromer (NTI, Fig. 5), can reverse this right shift and improve the analgesic response. Furthermore, we show that mice with a disruption of the DOR do not show a right shift in antinociception after the cocktail of methadone plus NTB (Fig. 5), suggesting that the effect of NTB is mediated by the DOR.

The studies here were not designed to examine the mechanism(s) underlying opioid tolerance. Rather, they were designed with the specific goal of determining the functional role of the DOR/MOR heteromer in analgesia. This is not a trivial distinction but an important one. Specifically, if one wishes to utilize the DOR/MOR heteromer as an analgesic target, it is critical to know whether one should be seeking agonists or antagonists. There is currently no consensus on this question, since previously there has not been a way to selectively activate or inactivate DOR/MOR heteromers without affecting either DOR and/or MOR homomers. Here, we used methadone as our probe analgesic, specifically because it promotes MOR endocytosis and recycling and would, therefore, not be predicted to cause many of the other MOR-mediated compensatory changes that occur with treatment with drugs such as morphine which do not internalize the receptor. This fact is evidenced by the small non significant shift in the EC_50_ in response to chronic methadone. More importantly, we could then use NTB, in conjunction with methadone, to stabilize DOR/MOR heteromers without affecting signaling from MOR homomers. In this way, we could selectively assess the role of the DOR/MOR heteromer in analgesia in response to a MOR agonist, thereby bypassing any contribution from DOR homomers since methadone has a low, µM affinity for DOR [19].

All signs from our studies here point to an anti-analgesic role of the DOR/MOR heteromer, at least for thermal nociception, which was measured in this study. We determined that the anti-analgesic effect produced by the combination of methadone plus NTB was dependent on the presence of DORs, since it was eliminated in the DOR knock out mice (Fig. 5A). Furthermore, we demonstrated that the anti-analgesic effect was reversed either by promoting degradation of DORs (Fig, 3C, Fig. 4) or by selectively blocking signaling only from the DOR/MOR heteromers but not MORs (Fig. 5). Thus, taken together, these data provide clear evidence for an anti-analgesic role of the DOR/MOR heteromer. In our saturation binding experiments we can not distinguished whether [^3^H]-DPDPE is bound to DOR homomers or DOR/MOR heteromers in the spinal cord membranes. Therefore, we can not rule out an upregulation of DOR homomers as well after chronic treatment with methadone plus NTB. However, the fewer DOR binding sites observed after treatment with methadone is most likely due to downregulation of DOR/MOR heteromers only, since methadone has a low affinity for DOR and does not promote DOR endocytosis [10]. Given these results, one might then expect that circumstances that promote upregulation of the DOR/MOR heteromer would manifest behaviorally as analgesic “tolerance”, defined as a reduced response to the same dose of drug. Previously, several lines of evidence have implicated an anti-analgesic role for the DOR in opioid tolerance. For example, morphine tolerance can be attenuated by antisense knock down of DOR [20], by genetic depletion of DOR [21], or by antagonists at DOR [11], [22], [23], [24], [25], [26], [27]. These findings were hard to reconcile, since DOR agonists themselves are antinociceptive. However, if one proposes that a subset of DOR, the DOR/MOR heteromers for example, are pronociceptive rather than antinociceptive, both the analgesia produced by DOR agonists (acting on DOR homomers) and the reduced tolerance produced by disruption of DOR signaling (acting on DOR/MOR heteromers) can be reconciled.

There continues to be much interest in elucidating the contribution of MOR and DOR, and by extension the DOR/MOR heteromer, to analgesia and the development of analgesic tolerance to opiate drugs in the hope of developing better therapeutics for the treatment of chronic pain. For example, recently, it was proposed that the co-degradation of MOR and DOR is responsible for morphine tolerance [8]. In effect, this study proposed an analgesic role for the DOR/MOR heteromer that is decreased due to heteromer degradation. However, this seems unlikely, since co-degradation of MOR and DOR would not be expected to occur under conditions where the MOR (and/or DOR) is not endocytosed (and therefore not degraded), such as in the presence of morphine [28], [29], [30], which drives endocytosis of neither the MOR nor the DOR. In fact, our studies here suggest just the opposite that the DOR/MOR heteromer has an anti-analgesic role, and that manipulations that cause upregulation of this target would promote “tolerance” revealed here as a right shift in dose response.

Morphine treatment may be one way to stabilize the DOR/MOR heteromer, since it does not promote endocytosis of either MOR or DOR. Indeed, recently it was shown that the DOR/MOR heteromer is upregulated after chronic morphine treatment [31]. Thus, upregulation of the DOR/MOR heteromer may be one factor that contributes to the development of morphine tolerance, though clearly it is not the only one. Even though it may be only one of a multitude of changes that contribute to morphine tolerance, it could be a clinically relevant one. Rotational therapy or opioid switching from morphine (which does not promote endocytosis of MOR or DOR) to methadone (which does promote endocytosis of both MOR and DOR/MOR) is a common clinical practice in patients as a means to delay tolerance [32], [33]. The biological mechanism by which rotational therapy functions remains unknown. Furthermore, a single dose of methadone is not sufficient to reverse tolerance to morphine suggesting, once again, that there are multiple distinct mechanisms contributing to morphine tolerance. However, it is intriguing to speculate that upregulation of the DOR/MOR heteromer may be one of the many molecular mechanisms mediating tolerance to morphine, and is one that can be reversed through rotational therapy. That being said, it is also clear that chronic morphine treatment causes many MOR mediated adaptive changes, other than merely DOR/MOR upregulation [34], which cannot be reversed with a single dose of methadone. Again the studies here were not designed to elucidate the mechanisms underlying morphine tolerance. Rather more specifically we wished to determine the contribution of the DOR/MOR heteromer to antinociception. Our findings that the DOR/MOR heteromer is anti-analgesic merely suggests that upregulation of this target could be contributing to analgesic tolerance. See the supplementary discussion section for additional discussion on this point (Discussion S1).

One proposed mechanism for the modulatory effect of DOR ligands on MOR-mediated analgesia and, by extension, tolerance is direct allosteric modulation of MOR agonist binding by the presence of DOR agonist/antagonist [5], [9], [11]. This allosteric modulation was postulated to be a consequence of action of the drugs in combination on DOR/MOR heteromers. However, it is also possible that the DOR antagonists used in the *in vivo* studies to block morphine tolerance are actually antagonizing the DOR/MOR heteromer rather than enhancing signaling from the DOR/MOR heteromer. In this case, the choice of ligand and its dose could be critical. For example, the DOR selective antagonist NTI efficiently blocks both the signaling and endocytosis of the DOR/MOR heteromers, whereas NTB only blocks endocytosis [10]. For example, here we show that adding NTB produces a profound right shift in methadone analgesia (Fig. 2) that was actually reversed by NTI (Fig. 5), even though both NTB and NTI are DOR antagonists. This is an example of “biased antagonism” with functional relevance for pain.

Our hypothesis of an anti-analgesic role for the DOR/MOR heteromer raises the question of how MOR and DOR/MOR activity, respectively, achieve opposite effects on analgesia. Intriguingly, DOR/MOR heteromers have been shown to switch their coupling from G_i_ to G_z_ [7], [35], [36]. Additionally, DOR/MOR heteromers have been shown to use beta-arrestin-2 to change the spatio-temporal dynamics of ERK phosphorylation [37]. In addition, beta-arrestin-2 KO mice show attenuated development to morphine tolerance compared to wild type mice [38], suggesting that signaling through beta-arrestin-2 contributes directly somehow in the development of analgesic tolerance. By extension, we speculate that enhanced signaling of the DOR/MOR heteromer to beta-arrestin-2 could contribute to tolerance, and would do so even in the presence of “normal” signaling from MOR homomers.

It is of particular relevance that any reduction in analgesia produced by methadone plus NTB was reversed, within 24 hours, by treatment with methadone alone. This suggests that reduced analgesia to methadone plus NTB is mediated solely by a mechanism(s) that is rapidly reversed by a single methadone exposure – which adaptive responses in the circuit should not be. This result, taken together with our findings that the right shift in the ED_50_ produced by the methadone cocktail is DOR dependent, and that it is reversed rather than exacerbated by another DOR antagonist with a different ligand bias (NTI), also strongly imply that it is not merely some off target effect that is responsible for the reduced antinociception shown in this study. See the supplementary discussion section for additional discussion on this point (Discussion S1).

Until there are agonists and/or antagonists selective for the heteromer versus the MOR and/or DOR homomers, we may not fully understand the functional contribution of the MOR, DOR and DOR/MOR heteromer under various physiological conditions. This is especially relevant since so many different stimuli have been shown to upregulate DOR – and possibly DOR/MOR function – including chronic opioid treatment, chronic inflammatory pain, stress, and ethanol consumption (for review see [39], [40]). Furthermore, it is possible that some of these stimuli upregulate antinociceptive DOR or DOR/MOR while others upregulate anti-analgesic DOR or DOR/MOR depending on the cells, circuits and signal transduction pathways that are active. However, here we show that at least under certain circumstances, the DOR/MOR heteromer opposes the analgesic effects of the MOR homomer. Thus, antagonists that selectively block activity of the DOR/MOR heteromer but not the MOR homomer could be powerful tools to use in conjunction with existing opioid analgesics for the treatment of chronic pain.

## Supporting Information

Figure S1 **DOR antagonist NTB combined with MOR agonist methadone changes the trafficking properties of the MOR, without affecting signaling of the receptor** ***in vitro*** **.** A) NTB blocks the endocytosis of DOR/MOR heteromers but not MOR homomers in response to methadone. HEK293 cells co-expressing FLAG-MOR and HA-DOR were surface-biotinylated and were either left untreated or pretreated with 1 µM of NTB 20 minutes prior to treatment with 1 µM of methadone for additional 30 minutes. MORs and DOR/MORs were selectively resolved by serial immunoprecipitation (see methods). “Total” shows the signal of the biotinylated receptors in cells after the initial labeling and without further manipulations; “strip” refers to biotinylated cells that reacted to gluthatione and demonstrates the efficiency with which biotin was cleaved from receptors. Both are internal controls within each experiment. Blots are representative of 3–5 independent experiments. B) Cells co-expressing DOR and MOR were pretreated with increasing concentration of the DOR antagonist NTB (closed squares) or with the MOR antagonist CTAP (open squares) for 20 minutes. Intracellular calcium release due to chimeric Δ6-Gqi4-myr activation was measured in a Flex Station apparatus after stimulation with a fixed concentration of methadone (1 µM). Data represents mean ± SEM; n = 3–5 experiments carried out in triplicate. C) MORs are stable while DOR/MOR are degraded after endocytosis in response to methadone. Cells co-expressing FLAG-MOR and HA-DOR were surface-biotinylated then were left untreated or were treated with 1 µM of methadone for 30, 60 and 120 minutes prior stripping. MORs and MOR/DORs were selectively resolved by serial immunoprecipitation (see methods). Blots are representative of 4–10 independent experiments.
(TIF)

Figure S2 **Saline injections do not have an effect on methadone antinociception.** Antinociception to escalating doses of methadone was measured in naïve wild type mice on day 1 (closed square). ED_50_ values were calculated via linear regression analysis and 95% confidence intervals are as follows: Day1, 3.5 (3.0–4.3) mg/Kg. On days 2, 3, 4 and 5, mice were injected s.c. once daily with saline. On day 6 (open circles) antinociception to methadone was measured again with ED_50_ values and 95% confidence intervals 3.6 (2.2–6.8) mg/Kg. Data represents mean ± SEM; n = 10 mice.
(TIF)

Figure S3 **Dose 0.1 mg/Kg of DOR antagonist naltrindole (NTI) is sufficient to block DOR-mediating mechanical sensitivity to SNC80 but allows MOR-mediating thermal antinociception to methadone.** A) Acute antinociceptive response was measured by tail-flick in C57/BL6 wild type mice after escalating doses (s.c.) of methadone alone (closed squares) or in combination with NTI (0.1 mg/Kg; open squares); n = 8 mice in both groups. Insert (A) shows acute methadone antinociception in the presence of different doses of NTI (1 mg/Kg, 0.5 mg/Kg and 0.1 mg/Kg); n = 8 mice for each NTI concentration. B) Acute mechanical sensitivity was measured by von Frey assay in C57/BL6 wild type mice after 20 mg/Kg of SNC80 (s.c.) alone or in combination with 0.1 mg/Kg of NTI. Data represents mean ± SEM; n = 8 mice per concentration. (Unpaired-t test, p = 0.012).
(TIF)

Discussion S1 **Here please find additional discussion.**
(DOCX)
