# Supplementary figures and images for: Anti-Analgesic Effect of the Mu/Delta Opioid Receptor Heteromer Revealed by Ligand-Biased Antagonism
Source: PLoS One. 2013 Mar 15;8(3):e58362. doi: 10.1371/journal.pone.0058362 (PMC3598907; doi:10.1371/journal.pone.0058362)

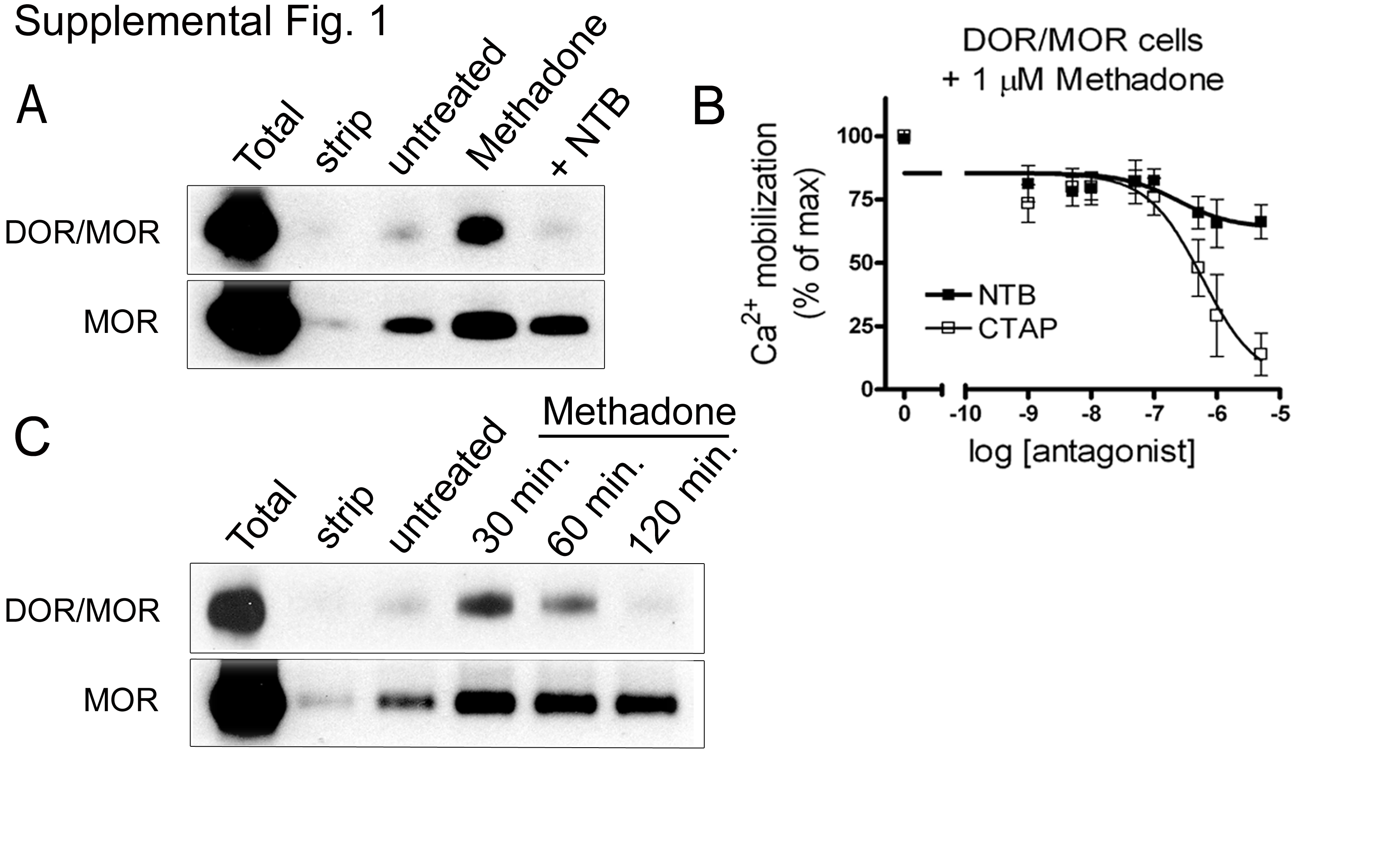

Supplement: Figure S1 — DOR antagonist NTB combined with MOR agonist methadone changes the trafficking properties of the MOR, without affecting signaling of the receptor in vitro . A) NTB blocks the endocytosis of DOR/MOR heteromers but not MOR homomers in response to methadone. HEK293 cells co-expressing FLAG-MOR and HA-DOR were surface-biotinylated and were either left untreated or pretreated with 1 µM of NTB 20 minutes prior to treatment with 1 µM of methadone for additional 30 minutes. MORs and DOR/MORs were selectively resolved by serial immunoprecipitation (see methods). “Total” shows the signal of the biotinylated receptors in cells after the initial labeling and without further manipulations; “strip” refers to biotinylated cells that reacted to gluthatione and demonstrates the efficiency with which biotin was cleaved from receptors. Both are internal controls within each experiment. Blots are representative of 3–5 independent experiments. B) Cells co-expressing DOR and MOR were pretreated with increasing concentration of the DOR antagonist NTB (closed squares) or with the MOR antagonist CTAP (open squares) for 20 minutes. Intracellular calcium release due to chimeric Δ6-Gqi4-myr activation was measured in a Flex Station apparatus after stimulation with a fixed concentration of methadone (1 µM). Data represents mean ± SEM; n = 3–5 experiments carried out in triplicate. C) MORs are stable while DOR/MOR are degraded after endocytosis in response to methadone. Cells co-expressing FLAG-MOR and HA-DOR were surface-biotinylated then were left untreated or were treated with 1 µM of methadone for 30, 60 and 120 minutes prior stripping. MORs and MOR/DORs were selectively resolved by serial immunoprecipitation (see methods). Blots are representative of 4–10 independent experiments. (TIF) [file pone.0058362.s001.tif]

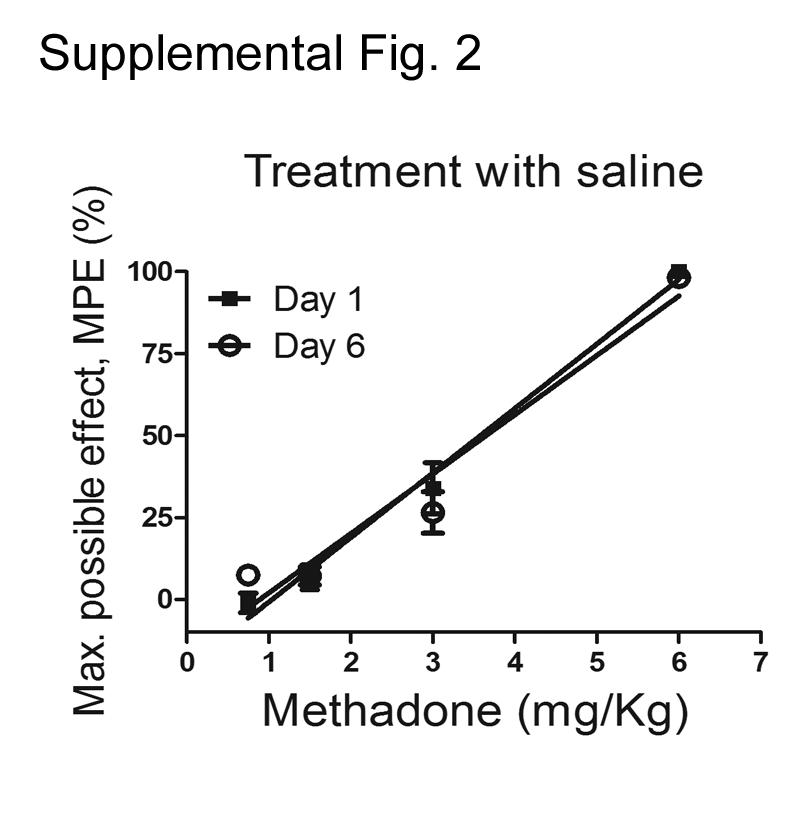

Supplement: Figure S2 — Saline injections do not have an effect on methadone antinociception. Antinociception to escalating doses of methadone was measured in naïve wild type mice on day 1 (closed square). ED50 values were calculated via linear regression analysis and 95% confidence intervals are as follows: Day1, 3.5 (3.0–4.3) mg/Kg. On days 2, 3, 4 and 5, mice were injected s.c. once daily with saline. On day 6 (open circles) antinociception to methadone was measured again with ED50 values and 95% confidence intervals 3.6 (2.2–6.8) mg/Kg. Data represents mean ± SEM; n = 10 mice. (TIF) [file pone.0058362.s002.tif]

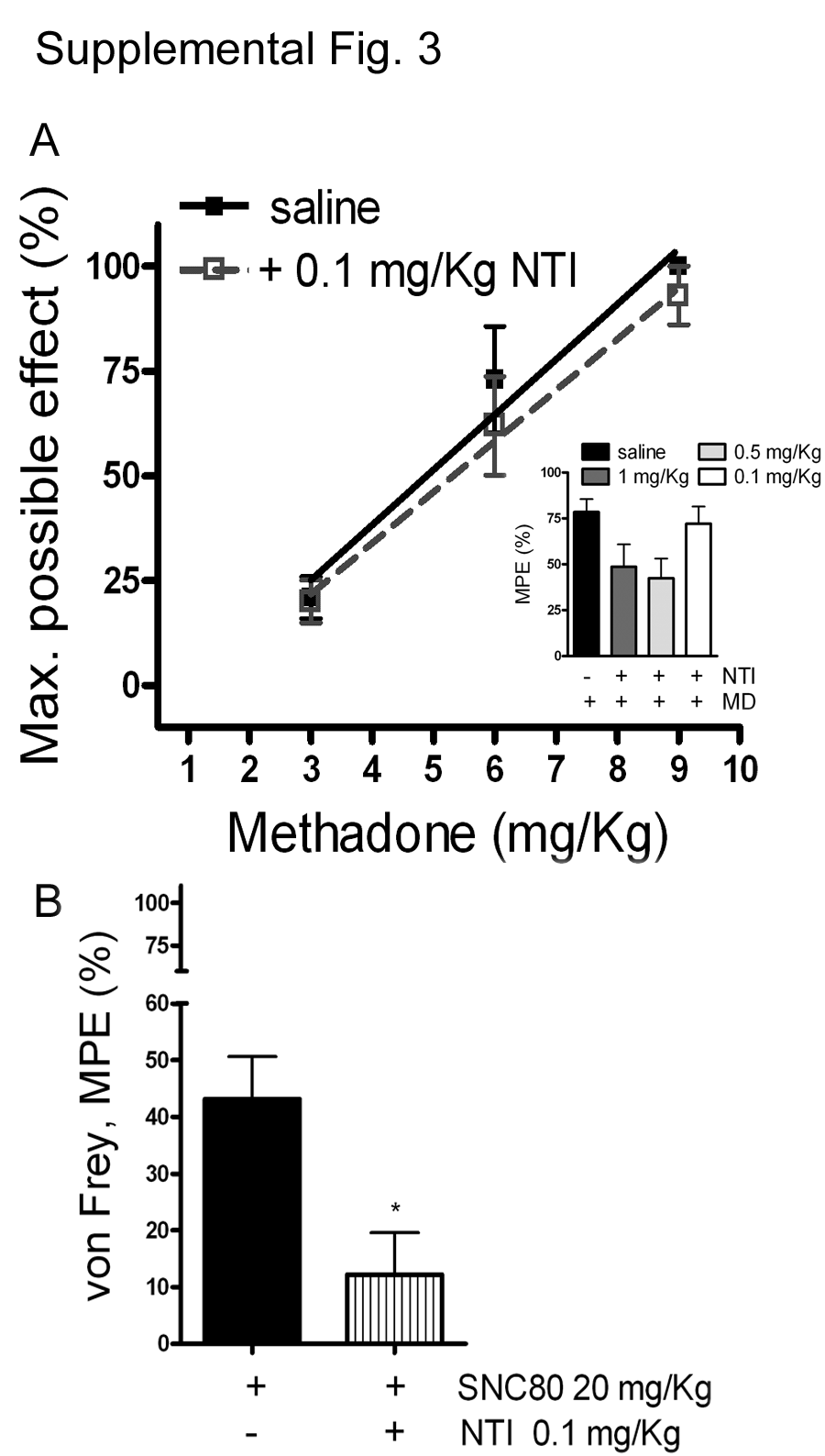

Supplement: Figure S3 — Dose 0.1 mg/Kg of DOR antagonist naltrindole (NTI) is sufficient to block DOR-mediating mechanical sensitivity to SNC80 but allows MOR-mediating thermal antinociception to methadone. A) Acute antinociceptive response was measured by tail-flick in C57/BL6 wild type mice after escalating doses (s.c.) of methadone alone (closed squares) or in combination with NTI (0.1 mg/Kg; open squares); n = 8 mice in both groups. Insert (A) shows acute methadone antinociception in the presence of different doses of NTI (1 mg/Kg, 0.5 mg/Kg and 0.1 mg/Kg); n = 8 mice for each NTI concentration. B) Acute mechanical sensitivity was measured by von Frey assay in C57/BL6 wild type mice after 20 mg/Kg of SNC80 (s.c.) alone or in combination with 0.1 mg/Kg of NTI. Data represents mean ± SEM; n = 8 mice per concentration. (Unpaired-t test, p = 0.012). (TIF) [file pone.0058362.s003.tif]
