# Supplementary material for: Anti-Analgesic Effect of the Mu/Delta Opioid Receptor Heteromer Revealed by Ligand-Biased Antagonism
Source: PLoS One. 2013 Mar 15;8(3):e58362. doi: 10.1371/journal.pone.0058362 (PMC3598907; doi:10.1371/journal.pone.0058362)
Supplement: Discussion S1 — Here please find additional discussion. (DOCX) [file pone.0058362.s004.docx]

**Supplemental Discussion S1**

While we favor the hypothesis that stabilization of anti-analgesic DOR/MOR heteromers by NTB produces the observed “tolerance” (reduced ED_50_ to methadone), it is possible that NTB is exerting its effects somewhere else in antinociceptive circuit(s) that modulates nociception. For example, release of endogenous cholecytokinin (CCK) negatively modulates opioid responses [1,2], and upregulation of CCK, which in turn negatively modulates opiate neurotransmission by activating CCK2 receptors [3] is thought to be one homeostatic adaptation that contributes to opioid tolerance. In fact, opioid agonists can directly stimulate the release of CCK, and this effect is dependent on the presence of DOR. Thus, one can imagine that chronic inhibition of DOR by NTB might lead to compensatory enhancements in CCK release that translate into reduced analgesia to methadone. However, if this were the case, we would expect all DOR antagonists to produce this effect. However, NTI actually reverses the tolerance induced by NTB plus methadone (Fig. 5C). Another possible mechanism by which NTB could be producing tolerance could be tied to the observation that NMDA receptor antagonists can inhibit the development and/or acquisition of tolerance [4]. One enantiomer of methadone binds with low affinity to NMDA receptors and functions as a non-competitive antagonist [5,6]. Thus, it is possible that the presence of low doses of NTB might somehow modulate the antagonistic activity of this enantiomer of methadone on NMDA receptors, and thereby reduce its analgesic effect. However, if this were the case, we would expect these off target effects of NTB on the NMDA receptor to remain intact even in mice with a disruption of DOR. However, the ability of NTB to shift the ED_50_ of methadone to the right is abolished in DOR KO mice (Fig. 5A). Thus, although we can not completely rule out the possibility that effects of NTB on some other system (other than the DOR/MOR heteromer) is responsible for its ability to reduce analgesia, there are several additional lines of evidence to suggest otherwise. Specifically, chronic treatment with NTB alone (with no methadone) did not produce the 2.7x fold right shift in the ED_50_ of methadone that was seen with the opioid cocktail of methadone and NTB (see results section). In addition, homeostatic adaptations, including increased release of CCK and/or NMDA receptor function, would be unlikely to be reversed by a single treatment of methadone alone (see Fig. 3C). On the other hand, endocytosis and degradation of DOR/MOR heteromers could happen within hours [7].

**Supplemental References**

1. Pommier B, Beslot Fo, Simon A, Pophillat M, Matsui T, et al. (2002) Deletion of CCK2 Receptor in Mice Results in an Upregulation of the Endogenous Opioid System. The Journal of Neuroscience 22: 2005-2011.

2. Wiesenfeld-Hallin Z, Xu X-J, Hökfelt T (2002) The Role of Spinal Cholecystokinin in Chronic Pain States. Pharmacology & Toxicology 91: 398-403.

3. Noble F, Fournie-Zaluski MC, Roques BP (1996) Opposite role of [delta]1- and [delta]2-opioid receptors activated by endogenous or exogenous opioid agonists on the endogenous cholecystokinin system: further evidence for [delta]-opioid receptor heterogeneity. Neuroscience 75: 917-926.

4. Trujillo K (2002) The neurobiology of opiate tolerance, dependence and sensitization: Mechanisms of NMDA receptor-dependent synaptic plasticity. Neurotoxicity Research 4: 373-391.

5. Callahan RJ, Au JD, Paul M, Liu C, Yost CS (2004) Functional Inhibition by Methadone of N-Methyl-d-Aspartate Receptors Expressed in Xenopus Oocytes: Stereospecific and Subunit Effects. Anesthesia & Analgesia 98: 653-659.

6. Ebert B, Thorkildsen C, Andersen S, Christrup LL, Hjeds H (1998) Opioid analgesics as noncompetitive N-methyl-aspartate (NMDA) antagonists. Biochemical Pharmacology 56: 553-559.

7. Marchese A, Paing MM, Temple BR, Trejo J (2008) G Protein-coupled receptor sorting to endosomes and lysosomes. AnnuRevPharmacolToxicol 48: 601-629.
